# Supported Biomembrane Systems Incorporating Multiarm Polymers and Bioorthogonal Tethering

**DOI:** 10.1021/acs.langmuir.4c00176

**Published:** 2024-05-20

**Authors:** Jesse A. Martin, Yue-Ming Li, M. Lane Gilchrist

**Affiliations:** ^†^Department of Chemical Engineering and the ^‡^Department of Biomedical Engineering, The City College of the City University of New York, 140th Street and Convent Avenue, New York, New York 10031, United States; § Chemical Biology Program, Memorial Sloan-Kettering Cancer Center, 1275 York Avenue, New York, New York 10065, United States

## Abstract

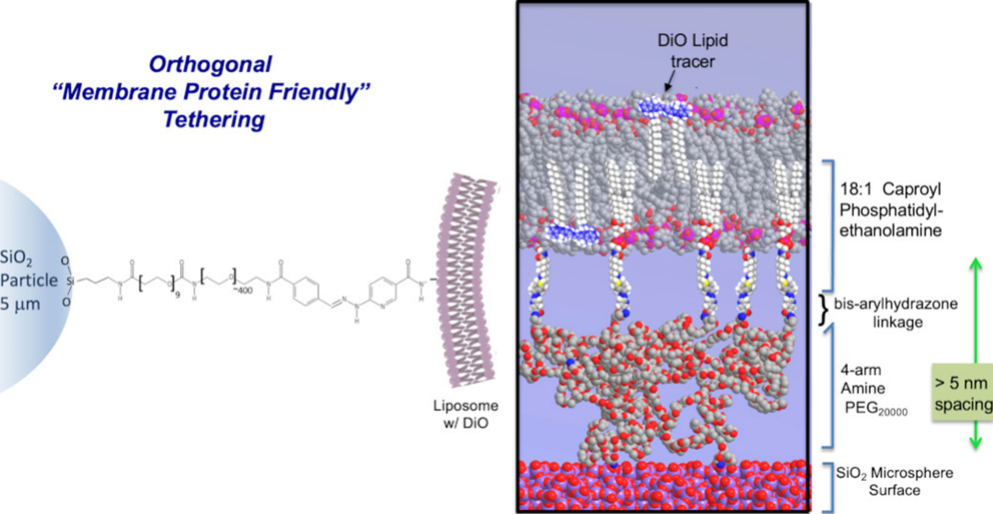

To functionalize interfaces with supported biomembranes and membrane proteins, the challenge is to build stabilized and supported systems that mimic the native lipid microenvironment. Our objective is to control substrate-to-biomembrane spacing and the tethering chemistry so proteoliposomes can be fused and conjugated without perturbation of membrane protein function. Furthermore, the substrates need to exhibit low protein and antibody nonspecific binding to use these systems in assays. We have employed protein orthogonal coupling schemes in concert with multiarm poly(ethylene glycol) (PEG) technology to build supported biomembranes on microspheres. The lipid bilayer structures and tailored substrates of the microsphere-supported biomembranes were analyzed via flow cytometry, confocal fluorescence, and super-resolution imaging microscopy, and the lateral fluidity was quantified using fluorescence recovery after photobleaching (FRAP) techniques. Under these conditions, the 4-arm-PEG_20,000_-NH_2_ based configuration gave the most desirable tethering system based on lateral diffusivity and coverage.

## Introduction

Supported lipid bilayer (SLB) technologies have been implemented to probe the function of biomedically relevant membrane proteins in controlled lipid microenvironments.^1^ There are various biomembrane tethering techniques utilizing hydrophilic poly(ethylene glycol) (PEG) molecules as a tethering moiety, cross-linking the substrate surface to a membrane-associated molecule.^3^

By controlling the molecular weight of the PEG molecules, one can potentially regulate the size of the separation between the substrate and the supported bilayer. Thus far, tether-supported biomembranes grafted onto surfaces have been limited primarily to single-chain linkers with molecular weights below 5000, shown to form PEG “brush” or “dense brush” regimes with relatively high grafting densities on particles and interfaces.^4^ To our knowledge, multiarm PEG tethers that have central three-dimensional (3D) symmetry have not yet been utilized and presumably would yield tether structures with inherent voids of substantial size adjacent to grafted multiarm polymers in the tethered biomembranes.^5^ Given that some of the most biomedically interesting membrane proteins have large extramembrane domains that protrude beyond 5 nm, these systems require longer tether lengths and both bilayer-adjacent void space to provide nonperturbing microenvironments. Another major concern is to avoid inadvertent surface-to-protein cross-linking that would interfere with membrane protein function and mobility, giving rise to artifacts in functional analysis and assays. These two constraints lead to the need to implement chemoselective ligation coupled with multiarm PEG-to-biomembrane tethering to build new microenvironments for membrane proteins.

Microsphere-based systems, termed proteolipobeads (PLBs), allow for washing during synthesis and assays steps and facile imaging with easier handling, higher experimental throughput, and larger assay areas in comparison to planar SLB systems. Another major advantage is that each microsphere assembly serves as an analyzable unit in flow cytometric assays, much like an individual cell,^6^ enabling analysis of large numbers of assemblies and also purification based on fluorescence activated flow sorting, if warranted.^7^ A disadvantage of PLB systems is that effective tethering in PLB systems requires the implementation of a heterobifunctional bioconjugation strategy to prevent the formation of multimicrosphere assemblies during the bioconjugation. Furthermore, tethering conjugation surface densities cannot be manipulated as easily experimentally as they are commonly done on surfaces using Langmuir–Blodgett trough setups that can concentrate the tethers in lateral areas and achieve the PEG brush or dense brush regimes in a controlled fashion, if warranted. In solution with surface conjugations to particles as done in this study, the linear PEG tethers are expected to be conjugated as a mixture of brush and mushroom surface densities dictated by the magnitude of PEG-NH_2_-to-substrate interaction, as evidenced in other PEG-grafted particle studies.^4^ The scope of this study includes the use of multiarm PEG tethers that gives rise to tether configurations that are anticipated to maintain internal structure when grafted to the substrates, leading to large separations between substrate and biomembrane inner leaflets and inherent voids adjacent to the grafted multiarm PEGs.^5^ Given the bioconjugation constraints, imine chemoselective ligation provides a supported biomembrane tethering route that has not yet been implemented, to our knowledge, and bioconjugation reagents based on reactions between hydrazines and aldehydes are now commercially available.^8,9^ The major advantage of these moieties over other more well-established cross-linkers such as NHS esters is that the reactive groups are hydrolytically stable in water at these conjugation time scales and thus can be introduced into proteoliposomes prior to fusion. In this study, we formed bis-arylhydrazone tethering cross-links from biomembrane-linked aromatic hydrazines to surface PEG-aromatic aldehydes. This was done using amino-reactive S-HyNic (sulfo-succinimidyl 6-hydrazinonicotinate acetone hydrazone), converting free lipid headgroup amine groups to HyNic groups (aromatic hydrazine). Similarly, S-4FB (sulfo-succinimidyl 4-formylbenzoate, SFB) was reacted with substrate-surface PEG-amine groups, converting them to aromatic aldehyde (4-formylbenzamide (4FB)) groups. Aniline was used as a rate-enhancing nucleophilic catalyst.^9^ Prior to supported bilayer formation, we characterized the resulting PEG-aldehyde surface coverage with fluorescein-5-thiosemicarbazide (FTSC-5)^10^ labeling, combining flow cytometry and confocal laser scanning microscopy (CLSM). After using liposome fusion to construct PEG-tether-supported PLBs, we characterized their formation using CLSM and structured illumination super-resolution microscopy (SIMs) and probed the lateral mobility of the resulting supported bilayers with confocal fluorescence recovery after photobleaching (FRAP).

## Results and Discussion

**Figure 1:**
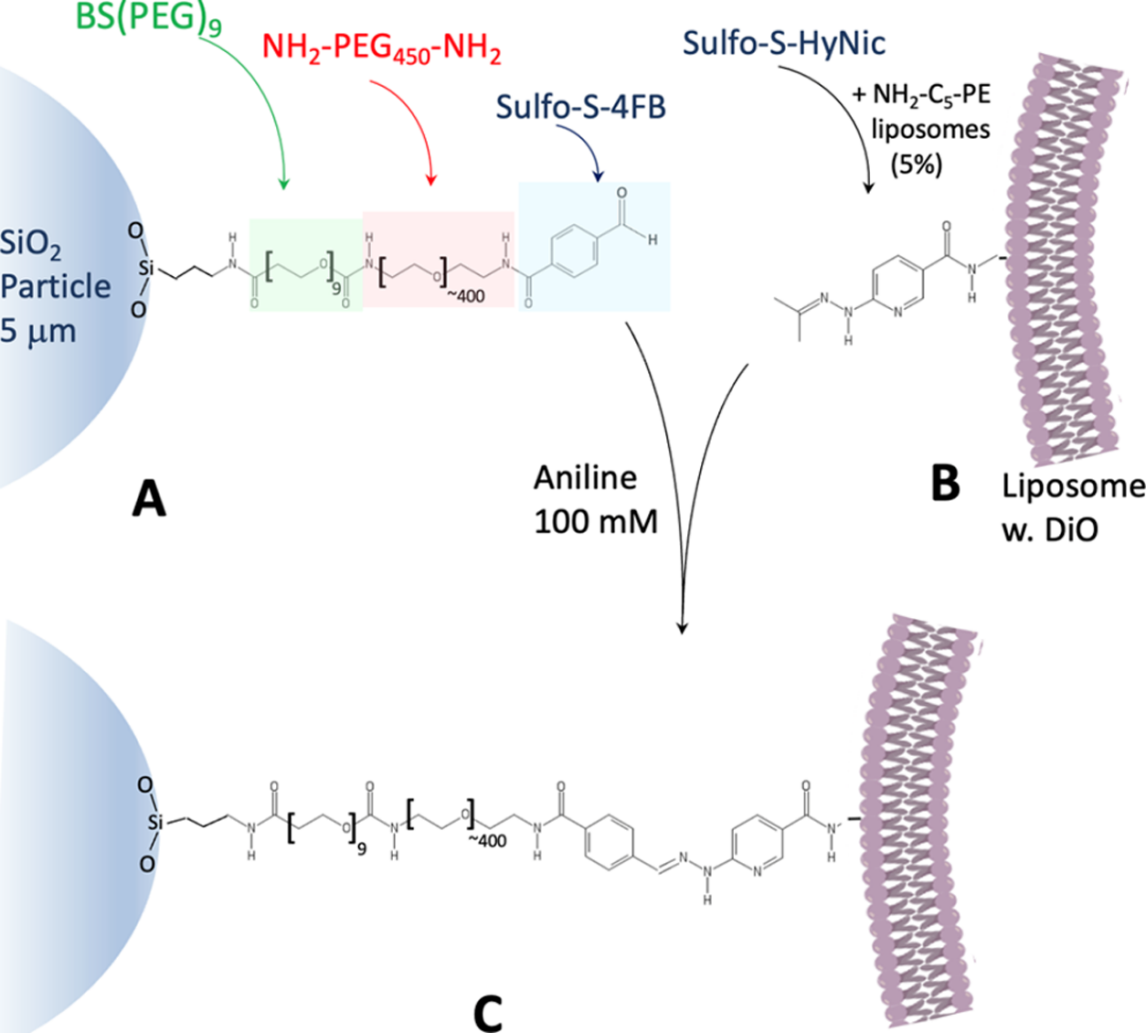
Schematic of supported biomembrane with protein orthogonal tethering via the bis-arylhydrazone linkage. We first couple the homobifunctional cross-linker BS(PEG)_9_ to functionalize the microspheres with (PEG)_9_-NHS. Then the PEG molecules are linked to the surface via PEG-amine to NHS coupling to functionalize the microsphere surfaces with the amine-PEG of interest. The surface PEG-amines are then reacted with Sulfo-S-4FB (4-formylbenzamide) to yield PEG-aldehydes at the surface available for forming bis-arylhydrazone linkages, as shown in (C). The liposomes (B) were functionalized with sulfo-S-HyNic at NH_2_-C5 phosphorylethanolamine lipids to enable the formation of tether-supported bilayers doped with the lipid reporter DiO at the silica microsphere surface.

The coupling concept at the core of this work is based on combining interchangeable multiarm amine-PEG spacers tethered with bis-arylhydrazone linkages to the lipid bilayer. As shown schematically in Figure 1, we first couple the homonobifunctional cross-linker BS(PEG)_9_ to functionalize the microspheres with (PEG)_9_-NHS. Then the PEG molecules are linked to the surface via PEG-amine to NHS coupling to functionalize the microsphere surfaces with the amine-PEG of interest. The surface amines are then reacted with Sulfo-S-4FB (4-formylbenzamide) to yield PEG-aldehydes at the surface available for forming bis-arylhydrazone linkages (Figure 1A). Liposomes doped with 5% NH_2_-C5-PE are functionalized with S-Hynic via NHS to amine coupling to yield liposomes functionalized with Hynic (6-hydrazinonicotinamide: Figure 1B), followed by removal of excess Sulfo-S-Hynic. Finally, the Hynic-terminated liposomes were fused with the PEG-aldehyde microspheres at 10-fold liposome total area to microsphere total area in the presence of aniline, a cross-linking rate accelerant (Figure 1C). This yields our intended interface structure, a tether-supported lipid bilayer linked to the surface with a PEG bis-arylhydrazone linkage. Any gaps in PEG coverage at the surface of the beads would be presumably conjugated with deactivated BS(PEG)_9_ cross-linkers with (PEG)_9_-carboxyl termination formed by hydrolysis of the NHS-containing BS(PEG)_9_ (3.58 nm spacer arm in linear configuration).

**Figure 2:**
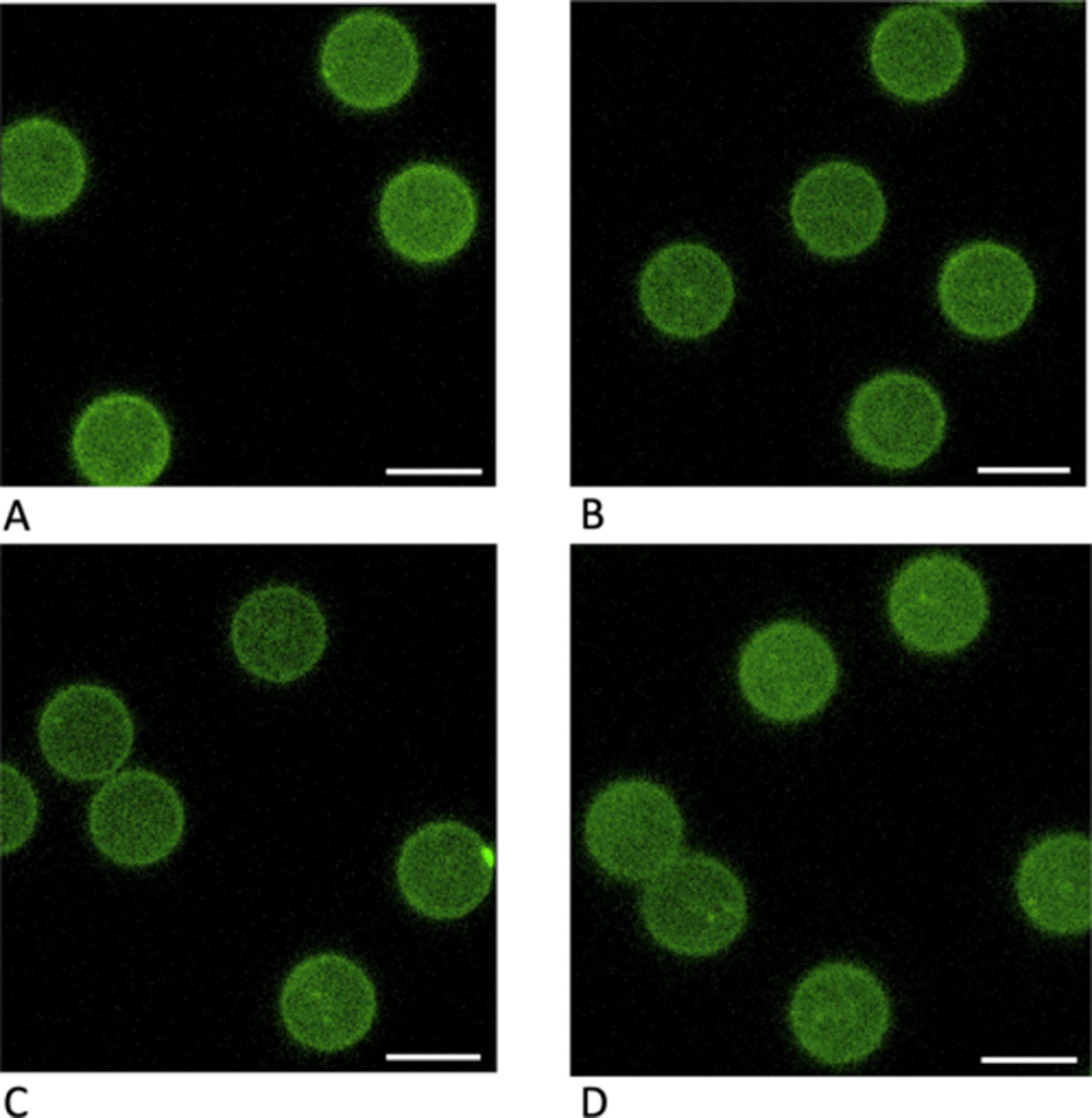
Confocal 3D hemispherical projections of surface PEG-4-FB aldehyde coverage visualized with (FTSC-5) hydrazine conjugation to the 4-FB. (A) Linear2k-PEG-amine conjugated with 4-FB. (B) Linear20k-PEG-amine conjugated with 4-FB. (C) 4arm20k-PEG-amine conjugated with 4-FB. (D) 8arm20k-PEG-amine conjugated with 4-FB. The FTSC fluorescein distribution is shown in green. Scale bar: 5 μm.

To examine the level and homogeneity of microsphere surface PEG-aldehyde functionalization with PEG-4FB, we employed the aldehyde-reactive labeling reagent fluorescein-5-thiosemicarbazide (FTSC-5). Flow cytometry and confocal microscopy were used to investigate the surface distribution of PEG-aldehydes through staining with FTSC-5. To probe the homogeneity of effective PEG tethering, we used confocal microscopy to assess the microsphere coverage in 3D. Figure 2 displays representative confocal hemispherical 3D projections of surface PEG-4-FB aldehyde coverage visualized with FTSC-5 hydrazine conjugation. Figure 2A and 2B are from linear2k-PEG-amine conjugated with 4-FB and linear20k-PEG-amine conjugated with 4-FB, respectively. Figure 2C and 2D are from 4arm20k-PEG-amine conjugated with 4-FB and 8arm20k-PEG-amine conjugated with 4-FB, respectively. Comparing these four images indicates striking homogeneity of PEG-aldehyde coverage, as intended. There is also no evidence of any significant gaps in coverage at the length scale examined by CLSM 3D reconstructions at this resolution. Analysis of the confocal intensity over a 2 μm line at the top of these projections yields a very similar apparent roughness parameter, as defined by Merzlyakov et al. in comprehensive fluorescence studies of tether-supported bilayers, defined as the ratio *r* = σ/*F*_0_, where *F*_0_ is the average fluorescence intensity and σ is the standard deviation of the intensity profile around the average value.^11^ The average roughness values were close in value and ranged from *r*_linear2k_ = 0.33 ± 0.06 to *r*_8arm20k_ = 0.46 ± 0.06 (see Supporting Data Figure S2). The roughnesses of linear2k, linear20k, and 4arm20k were not statistically significantly different in magnitude.

**Figure 3:**
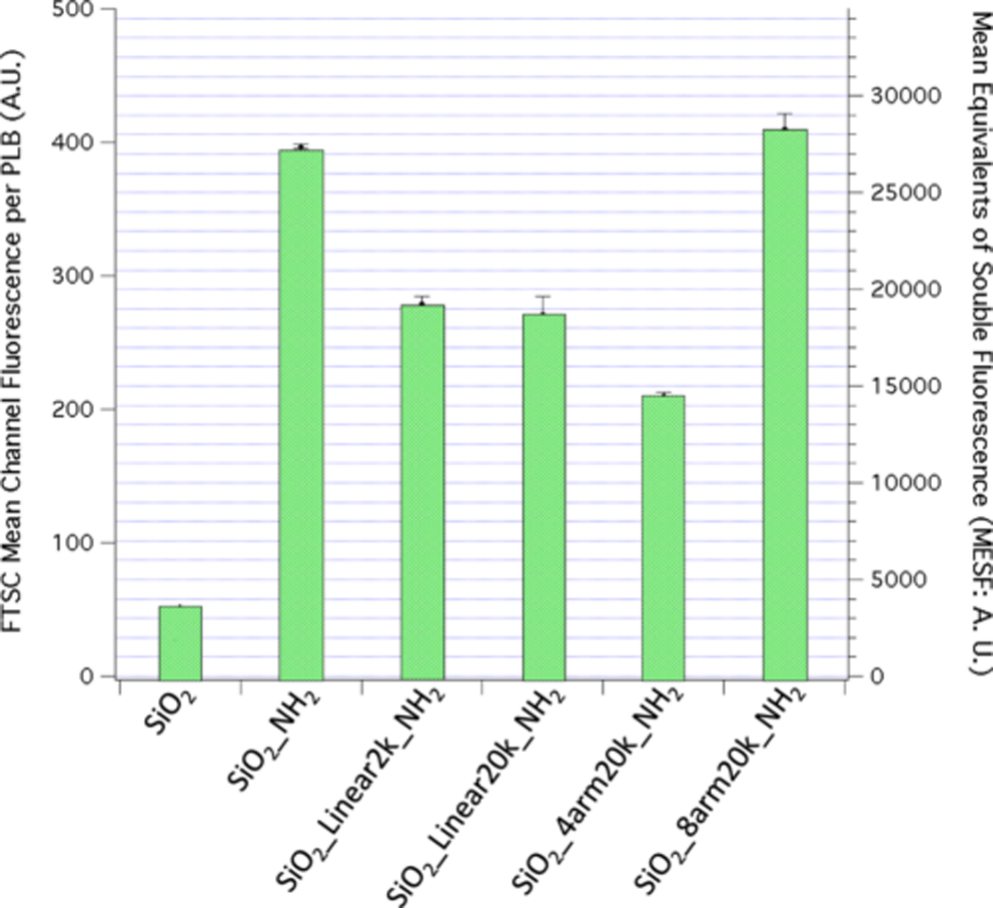
Flow cytometric analysis of microsphere substrate-surface PEG-aldehyde functionalization detected by fluorescent fluorescein-5-thiosemicarbazide (FTSC-5) hydrazine conjugation to form hydrazone linkages. Values are given ± standard error (σ/√*N*). The FTSC-5 mean channel fluorescence per microsphere was obtained from ∼10,000 microspheres from three independent samples.

To further examine the relative levels of surface aldehyde functionalization, we examined the samples with flow cytometry. The bar chart in Figure 3 displays the FTSC-5 mean channel fluorescence of our multiarm PEG-aldehyde-terminated substrates compared with plain silica control particles. For reference, we included amine-terminated silica particles labeled with 4-FB. The right axis gives an estimate of the approximate aldehyde surface coverage per square micron as assessed using Mean Equivalents of Soluble Fluorescence (MESF) quantitation methodology from Bangs Laboratories. This surface fluorescence estimation method is intended to provide a qualitative framework for assessing the levels of reactive moieties available for tethering the supported biomembrane. In addition, as a consistent, calibrated set of fluorescein MESF standards range in intensity over our workable range in the cytometer, we can compare mean channel fluorescence intensity from independent cytometry runs on separate days. However, there are major limitations that must be considered that compromise the utility for accurate molecular quantitation with this method. First and foremost, the microenvironment of the fluorescein in the MESF standards is a polymer matrix and not a fluorescein moiety dangling from a PEG conjugate in buffer solution, so a one-to-one molecular quantitation is not possible due to possible differences in fluorescence lifetime. Furthermore, in our surface labeling scenario, the readout of surface functionalization is expected to be compromised by fluorescein self-quenching if the spacing between the labeled sites is <5 nm, as shown in multiple studies and characterized carefully by Deka et al.^10^ Based on geometric arguments, we expect that the intensity levels of the case where 4-FB is directly conjugated to closely spaced surface amines in the SiO_2__NH_2_ case will give an apparent intensity level that does not accurately reflect the true higher levels of aldehyde coverage due to extensive self-quenching. However, based on the Flory radii of PEG in the mushroom regime and our examination of the diameters of the PEGs employed in this study with dynamic light scattering (DLS), we can begin to take into account the quenching.^12^ In contrast to Langmuir–Blodgett trough-based planar surface studies where grafting density can be tuned, it is likely that only low grafting densities in the PEG “mushroom” regime are accessible in microsphere surface PEG conjugation to form SLBs under these solvent conditions (phosphate-buffered saline (PBS) buffer: a “good solvent”; χ ∼ 0.5^5,13^). In essence, under these conditions, PEG excluded volume effects limit the density of grafting of the chains to the surface. Table 1 gives some reference data based on the Flory radius of individual arms of the PEG linkers in the study along with DLS sizing data for the multiarm polymers in solution. Using simple geometric arguments, we estimated the minimum amine separation distance for the PEG tethers. From this analysis and the ∼5 nm quenching threshold, we expect that the linear2k-PEG-aldehyde sites will experience some degree of fluorophore-to-fluorophore quenching (*R*_F,PEG2000_ ≈ 3.5 nm), as well as the 8arm20k-PEG (single arm *R*_F,PEG2500_ ≈ 3.7 nm; minimum amine separation distance ≈4.9 nm). The closely packed SiO_2__NH_2_ case, labeled directly with FTSC-5, would be very highly quenched. This would give rise to unquenched FTSC-5 intensity levels significantly higher than those reported in Figure 3 for these cases. Nonetheless, based on these spatial arguments, we expect that linear20k-PEG (*R*_F,PEG20,000_ ≈ 13.6 nm; minimum amine separation distance ≈13.6 nm) and 4arm20k-PEG (single arm *R*_F,PEG5000_ ≈ 6.0 nm; minimum amine separation distance ≈10.3 nm) are most likely not compromised by significant fluorophore-to-fluorophore quenching. Taking into account this probable quenching, in Figure 3, we note that the 4arm20k-PEG linker still gives the lowest aldehyde surface density of this series of tethers as detected by this method, with significantly lower degree of FTSC-5 labeling (*p* < 0.05) over the other three PEG linkers. If we consider the condition where the available surface is completely conjugated with the PEG-aldehydes, based on the circular area footprint of the linkers in the mushroom regime, we can estimate the available tethering density for each case. We outline these configurations in Figure S3 of the Supporting Data section. Neglecting surface roughness and assuming a circular packing fraction of 0.909 and no significant PEG overlap,^14^ we can estimate the (upper bound) relative available cross-linking site density per 100 nm^2^ area as 2.6 for linear2k, 0.6 for linear20k, 1.6 for 4arm20k, and 10.5 for 8arm20k. These values would be lower if we take into account that a definite unknown fraction of the surface-conjugated BS(PEG)_9_ NHS groups have hydrolyzed to give unreactive PEG_9_-carboxy-terminal moieties in the incubation period before the amine-PEGs are added for tethering.

**Table 1:** Summary of DLS Measurents in the Context of PEG Flory Radii

	estimated Flory radius (*R*_F_) single arm (mushroom) (nm)^a^	average diameter via DLS in PBS solution (2 × *R*_hyd_; nm)	estimated minimum amino separation distance in solution (nm)^b^
linear2k	3.5	nd	3.5
linear20k	13.6	13.6 ± 2.8	13.6
4arm20k	6.0	12.6 ± 4.8	10.3
8arm20k	4.0	8.5 ± 1.5	4.9

a From *R*_F_ = *a*_m_*n*^3/5^ with *a*_m_ = 0.39 nm.^2^ Linear2k was not detectable in the Malvern Zetasizer DLS, see Figure S1.

b Considering *R*_F_ for 1arm2k and 1arm20k and geometric considerations for terminal amino groups positioned as PEG arms radiating out from the center and positioned relative to the faces of a tetrahedron (4arm20k) or octahedron (8arm20k).

We have developed this method in the context of our recently published method to probe the levels of loading of the enzyme γ-secretase and its membrane-bound substrate in proteolipobeads for functional analysis.^15^ As we have used anti-FLAG-FITC to assess the PLB surface levels of γ-secretase substrate labeled with the FLAG epitope, a major potential issue we aim to address with the tethering methodology is the suppression of the nonspecific binding of the fluorescein isothiocyanate (FITC) antibody. We assessed this using cytometry at the same concentration of anti-FLAG-FITC antibody used in our previous work. We compared the nonspecific binding of three systems: (1) plain silica beads, (2) amine-terminated silica beads, and (3) silica bead labeled with BS(PEG)_9_-conjugated 4arm20k-PEG-NH_2_. The PEG-free amine-terminated silica beads yielded an undesirable 15.4-fold level of nonspecific binding of anti-FLAG-FITC over plain silica beads (silanol hydroxyl-terminated). However, the BS(PEG)_9_ linked 4arm20k-PEG-NH_2_ beads brought the nonspecific binding back down to near the level of the plain silica beads (*I*_silica_ = 1 (normalized); *I*_4arm20k-PEG-NH_2__ = 0.86), indicating that the use of our BS(PEG)_9_/multiarm PEG tethering strategy suppressed nonspecific binding to low levels in regions that ultimately would not contain supported biomembranes.

We now examine the PLBs formed from liposome fusion to give bis-arylhydrazone linkages between the aldehyde-PEGs on the surface and hydrazine-lipid headgroups in the supported bilayer. This fusion was carried out at 45 °C in 100 mM aniline in PBS buffer (depicted schematically in Figure 1C). The aniline has been shown to increase the rate of these imine cross-linking reactions as a nucleophilic catalyst in multiple studies, with rate enhancements over 3 orders of magnitude in some cases.^9^ In this tethering application, the presence of aniline was vital for the success of fusion, as these largely PEGylated surfaces are highly passivated to liposome fusion in general. Our trials to produce fusion were of unacceptably low quality and coverage without the rate enhancement of aniline and we did not go beyond visual inspection as the lipid signal was not good enough to adequately image (other negative control examples are shown in Figure S5 for reference).

**Figure 4:**
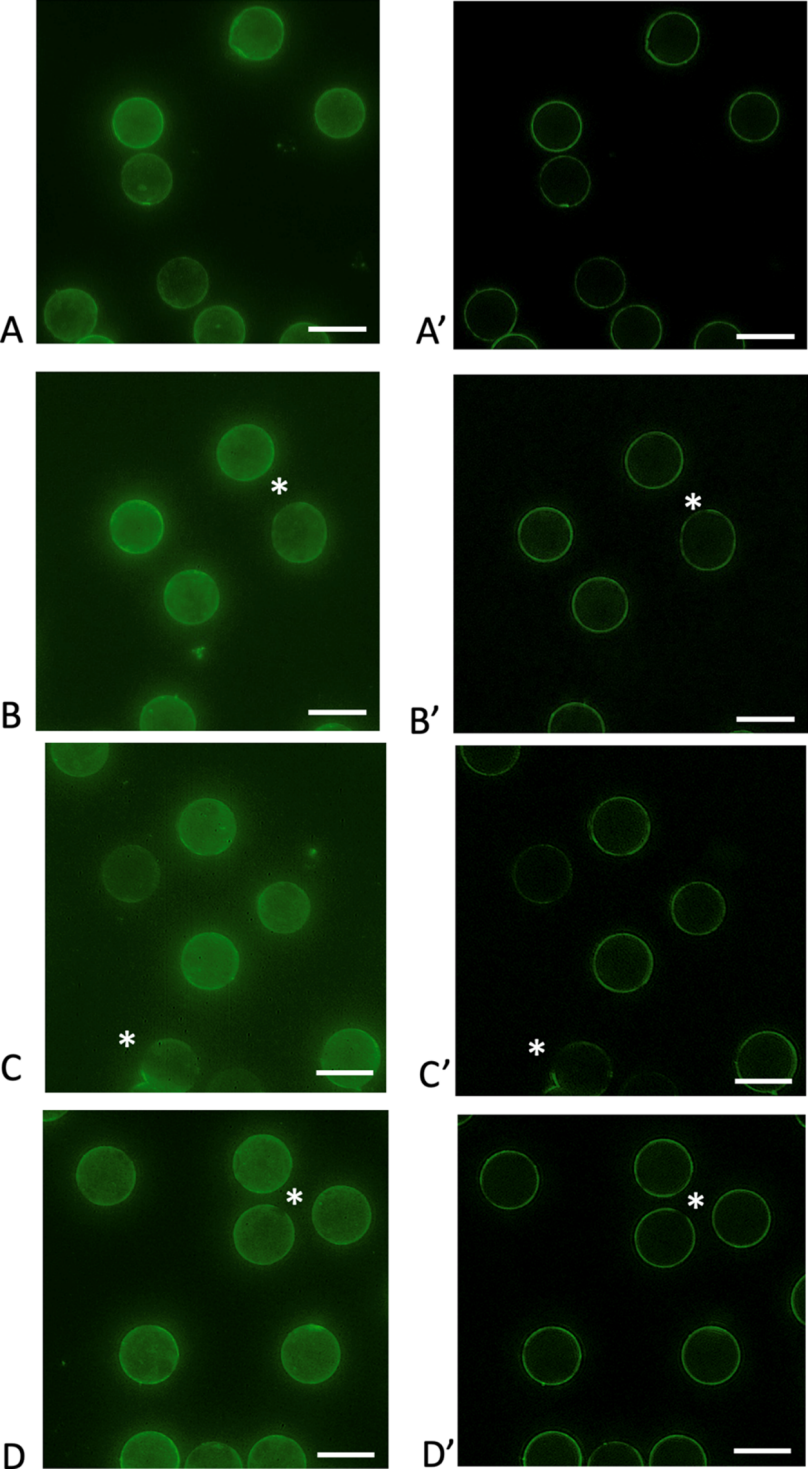
Representative 3D projections of structured illumination super-resolution microscopy of PEG-tether-supported proteolipobeads. Panels (A)–(D) were obtained from summed Z-projections of the SIMs Z stacks. (A, A′) Linear2k-PEG-amine conjugated with 4-FB. (B, B′) Linear20k-PEG-amine conjugated with 4-FB. (C, C′) 4arm20k-PEG-amine conjugated with 4-FB. (D, D′) 8arm20k-PEG-amine conjugated with 4-FB. The right panels (A′)–(D′) indicate the equatorial sections only. The white asterisks indicate representative visible inhomogeneities. The lipid reporter DiO (green) distribution is shown. Scale bar: 5 μm.

To probe the coverage and homogeneity of the structures formed, we performed CLSM and structured illumination super-resolution microscopy (SIMs) of the PEG-tether-supported PLBs. Figure 4 illustrates representative 3D projections reconstructed from structured illumination super-resolution microscopy of a hemisphere of PEG-tether-supported lipid bilayers. These representative reconstructions show mostly uniform supported bilayer structures with some surface inhomogeneities and gaps in supported biomembrane coverage that were not evidenced in the analysis of PEG-aldyhyde coverage via FTSC-5 (Figure 2).

**Figure 5:**
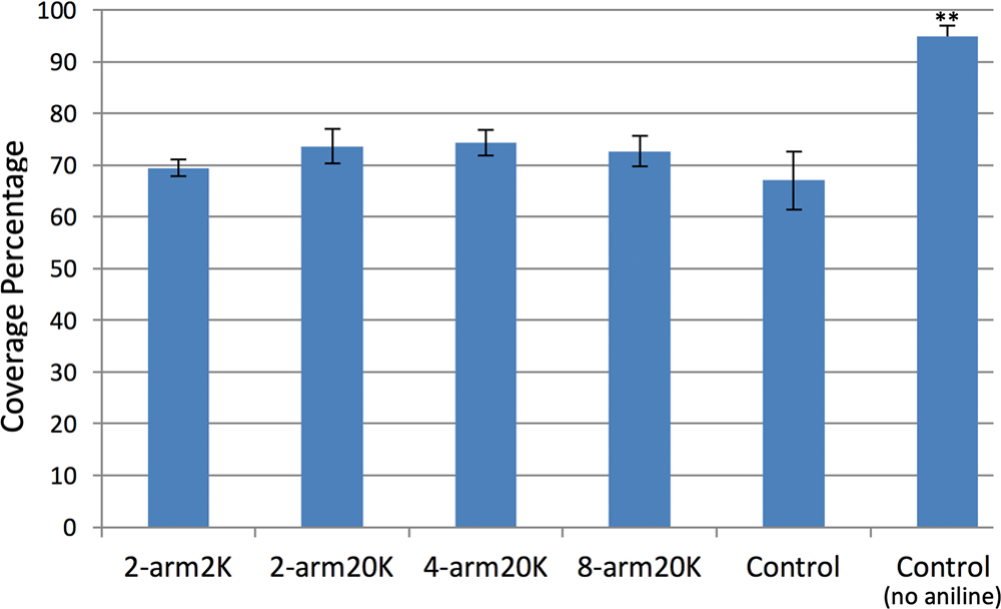
Coverage summarizes the CLSM supported membrane coverage percentage levels obtained under the PLB fusion conditions. The far right sample is the control (untethered) measured without 100 mM aniline present. Values are given ± standard error (σ/√*N*). Only the control (untethered) PLBs that did not contain aniline gave a statistically significant difference (*p* < 0.05; indicated with **).

An in-depth analysis of surface coverage and biomembrane defect levels was conducted on the basis of the 3D reconstructions of *N* = 20 randomly selected microspheres of each type by examining the CLSM lipobead structures obtained. These data are summarized in Figure 5. In the tethered cases, in the presence of 100 mM aniline at 45 °C, the coverage was greater than or equal to ∼70%. Closer examination of the supported bilayer SIMs imaging data reveals large gaps in coverage where PEGylation is most likely still present due to the lack of liposome adsorption or fusion. These regions appear to be consistent with damage induced by a combination of aniline interacting with the supported bilayer leading to solubilization in concert with the 45 °C incubation and collisions between microspheres and also collisions against the chamber walls and the highly disruptive air–water interface. The untethered control PLB sample that did not contain aniline yielded a coverage value of 94.9 ± 2.1% (data on the far right of Figure 5). With the exception of the aniline-free untethered control, the magnitude of coverages is considerably lower than we have seen in our previous studies involving PLB tethering with homobifunctional cross-linking from surface amines to lysines in anchor α-helical peptides using NHS-PEG_3000_-NHS (>90% for both tethered and untethered control PLBs).^16^ As discussed, we attribute this to a combination of factors: (1) fundamentally different conditions for fusion and cross-linker reactions (45 °C versus room temperature) and (2) the presence of aniline as a arylhydrazone ligation reaction accelerant at significant concentrations (100 mM).^9^

**Figure 6:**
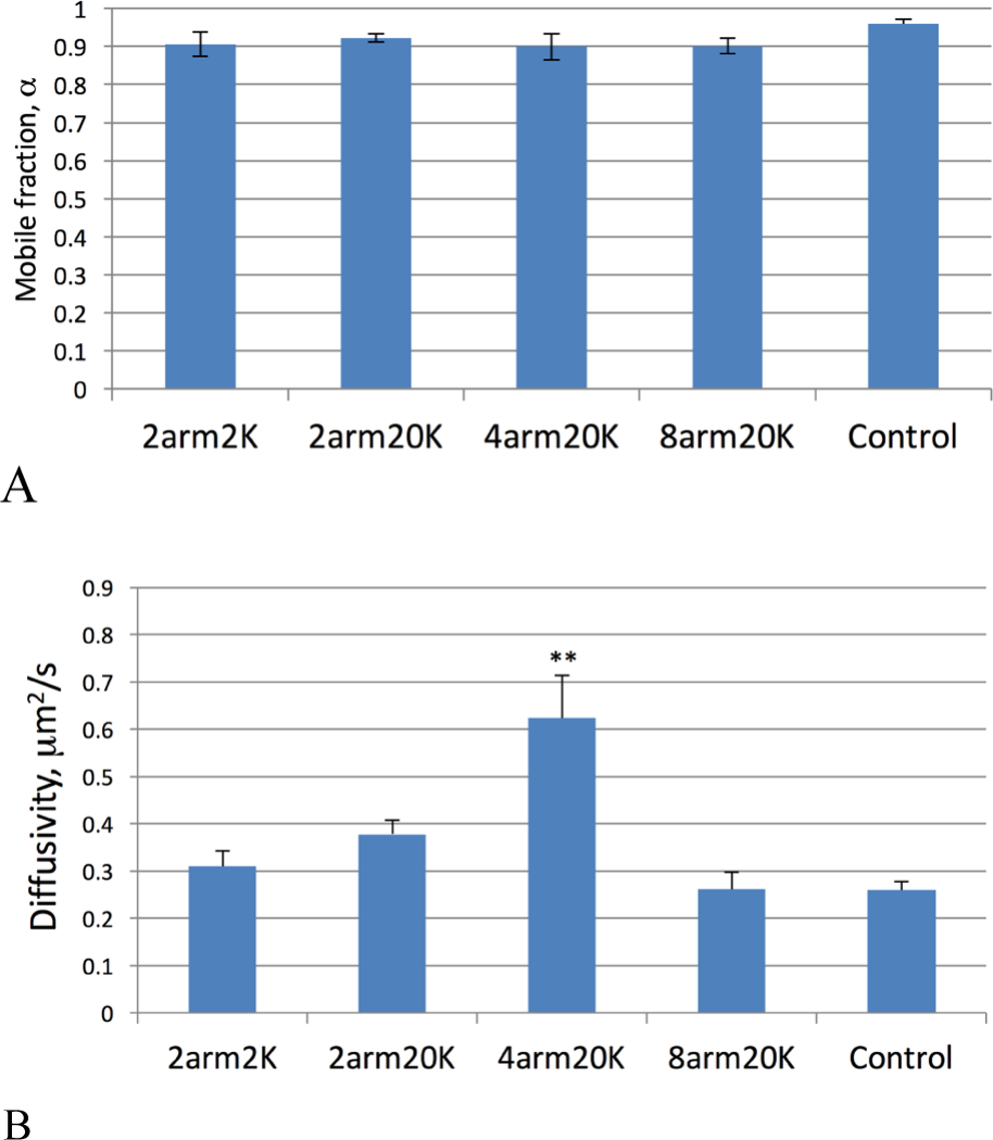
FRAP analysis of tether-supported lipid bilayer lateral mobility of the fluorescent reporter DiO. Panel (A) displays the mean value of the recovery mobile fraction, and panel (B) gives the effective diffusion coefficient. Values are given ± standard error (σ/√*N*). In panel (A), none of the average mobile fractions were found to be statistically different. In panel (B), only the 4arm20k case was statistically different from the others (*p* < 0.05; indicated with **).

We conducted confocal-based fluorescence recovery after photobleaching (FRAP) studies to characterize the lateral mobility of the PEG-tethered PLB supported biomembranes. The effective diffusion coefficient of the embedded DiO reporter, *D*_eff_, can be determined as we have done in previous studies.^16−18^ We also measured the average recovery fraction denoted as the mobile fraction, α. The results of FRAP measurements in the equatorial z section are tabulated in Figure 6. As seen in Figure 6A, the mobile fraction is near 0.9 without statistically significant differences (*p* > 0.05) for all of the cases examined, indicative of fluid and continuous supported lipid bilayers present in the photobleached region of interest.

The confocal-FRAP-derived effective diffusivities of the lipid reporter DiO are given in Figure 6B. The mean effective diffusion coefficients are not statistically different for the linear2k-PEG and linear20k-PEG samples (*p* > 0.05), with, respectively, values of *D*_eff,linear2k-PEG_ = 0.31 ± 0.03 μm^2^/s and *D*_eff,linear20k-PEG_ = 0.37 ± 0.03 μm^2^/s. However, for the 4arm20k-PEG sample, a greater than 85% increase in diffusivity over the average of the other three PEG cases was evidenced (**), with *D*_eff,4arm20k-PEG_ = 0.62 ± 0.09. The diffusivity in the 8arm20k-PEG sample was statistically significantly lower than that in linear20k and 4arm20k, with a value of *D*_eff,8arm20k-PEG_ = 0.25 ± 0.05 μm^2^/s. When taken in the context of the PEG-aldehyde coverages detected by FTSC-5 labeling (Figure 3), an important correlation is seen in the 4arm20k and 8arm20k tethering cases. The PEG tether with the lowest measured aldehyde surface density yielded the highest average effective diffusivity. Also, this linker would presumably give the highest average surface to biomembrane separation (single arm *R*_F,PEG5000_ ≈ 9 nm; total spacing >15 nm). Given that the levels of coverage and apparent surface roughness were similar in all of the PEG cases, our finding is that most likely the level of bilayer tethering density and spacing afforded by the 4arm20k-PEG linker yielded the best results for future studies that will incorporate membrane proteins. As it has been seen that the nature of the PEG tether length can disturb the lipid packing leading to changes in local diffusivity of reporters in supported membranes, as in the work of Socrier et al., it is difficult to make concrete determinations of the impact of the different PEG configurations.^19^ When measuring PEG–lipid conjugate diffusivity in supported membranes, it has been shown that the anchoring moiety matters most up until headgroup PEG crowding comes into play,^20^ and we will likely evidence this type of phenomena once membrane proteins are embedded. Within the limited scope of our study, it is difficult to go beyond a correlation and separate the PEG spacing from the simultaneous effect of the tethering density, but we aim to probe this further in future studies. At this point, this tethering architecture is ready for incorporation of membrane protein drug targets such as γ-secretase into the tethered PLBs.^18,21^ This includes endogenous lipid systems such as giant plasma membrane vesicles (GPMV) that preserve the plasma membrane composition to a large degree. Our architecture outlined here has ample microsphere-to-biomembrane spacing that will allow us to study membrane proteins with larger extramembrane domains using a biorthogonal tethering chemistry that will not give rise to unwanted protein conjugation that could affect function.

## Conclusions

In this work, we have outlined a new method to form tether-supported biomembranes using a novel biomembrane cross-linking chemistry that is designed to not perturb membrane protein components in the tethering process via undesired reactions. Including the particle modality allows for new ways to monitor intermediate steps of reactive tether surface density and homogeneity in the route to tethered biomembranes. Furthermore, we have integrated some of the new multiarm PEG tethering moieties in a modular format designed to provide ample spacings beyond the size of most extramembrane domains found in nature. In particular, since our ongoing work is directed at the highly biomedically relevant drug target γ-secretase (known to have a very large protruding subunit (∼7 nm)),^18,22^ in an atomic structure of human γ-secretase, this tethering system should be suitable for further studies of this system under conditions where substrate-to-protein interactions are minimized. We note that there is a trade-off in enhancing the coupling reaction with the addition of a substantial concentration of aniline that has a deleterious effect on the supported biomembrane integrity. This is the consequence of employing a commercially available coupling scheme that was originally intended for soluble protein bioconjugation, where the presence of aniline in significant amounts would serve as a rate enhancer that would not affect protein coupling. In general, aniline at this concentration would serve its function as a rate enhancer without affecting the soluble protein systems to be coupled and would be washed out in subsequent purification steps. In future studies with biomembranes, we will explore the use of substitute enhancers that would not likely perturb the supported bilayers during cross-linking. Nevertheless, this new method sets out into a new direction that could overcome the present tethering issues of spacing and deleterious surface-to-membrane protein interactions as well as undesired bioconjugation side reactions that could perturb membrane protein functional analysis.

## Experimental Section

### Materials

Amine-functionalized silica microspheres of 4.9 μm nominal size were purchased from Bangs Laboratories (West Bend, IN). 3,3′-Dioctadecyloxacarbocyanine perchlorate (DiO) and BS^3^ (bis(sulfo-succinimidyl)suberate) were obtained from Life Technologies, Inc. Fluorescein isothiocyanate (FITC), NHS-PEG_4_-biotin, and chloroform were obtained from Thermo Fisher Scientific Corp. (Chicago, IL). Fluorescein-5-thiosemicarbazide [FTSC-5] was obtained from AnaSpec Inc. (Fremont, CA). The lipids were obtained from Avanti Polar Lipids, Inc. (Alabaster, AL) and used without further purification. The study included 1-palmitoyl-2-oleoyl-*sn*-glycero-3-phosphocholine (POPC), cholesterol, and 1,2-dipalmitoyl-*sn*-glycero-3-phosphoethanolamine-*N*-(hexanoylamine) (16:0 caproylamine PE; NH_2_-C5-PE). The PEG-amines, denoted NH_2_-PEG_2000_-NH_2_ (linear2k), NH_2_-PEG_20,000_-NH_2_ (linear20k), 4-armPEG_20,000_-NH_2_ (4arm20k), and 8-armPEG_20,000_-NH_2_ (8arm20k) were obtained from Nanocs, Inc. (Boston, MA) and had purities of greater than 95% with polydispersity index (PDIs) < 1.08. We used Nunc Lab-Tek II Chambered Coverglass slides with a No. 1.5 borosilicate glass bottom in all of the studies.

### Tethered Substrate Preparation

Amine-functionalized silica microsphere substrates were titrated at ∼10 mg/mL into an aqueous solution of BS(PEG)_9_ at ∼1.33 mg/mL, for a total of ∼5 mg of substrate SiO_2_-NH_2_ particles per 2 mg of BS(PEG)_9_. This solution was then vortexed for 5–10 s and then mixed at room temperature for 30 min, well below the reported half-life for NHS esters at these conditions (∼1 h at pH 8, 25 °C).^23^ The beads were then pelleted and rinsed three times with PBS, resuspended at a concentration of 10 mg/mL, and titrated into a solution of NH_2_-PEG_2000_-NH_2_ (linear2k), NH_2_-PEG_20,000_-NH_2_ (linear20k), 4-armPEG_20,000_-NH_2_ (4arm20k), and 8-armPEG_20,000_-NH_2_ (8arm20k) for a final bead concentration of 2.5 mg/mL and a final PEG concentration of ∼20 mg/mL, this solution then mixed gently for 2 h at room temperature, rinsed with PBS as previously described, and resuspended at 10 mg/mL. Aldehyde functionalization involves titration of this PEG-NH_2_ functionalized bead suspension into a 0.2 mg/mL aqueous Sulfo-4-FB solution for a final Sulfo-4-FB concentration of 0.1 mg/mL and a final bead concentration of ∼3 mg/mL. This reaction is then gently mixed at room temperature for 1.5 h, then rinsed, as previously described. Negative controls were generated by incubating 1 mg of each sample of aldehyde-free, PEGylated beads in 1 mL of 20 mg/mL mPEG-SPA (MW 2k, Shearwater) for 1 h, mixing at room temperature. The mPEG-SPA caps the amino sites on the hydrazine-free, PEGylated substrates, preventing potential binding with aromatic hydrazines on the sulfo-S-hynic functionalized liposomes. In addition, we tested liposome fusion with microsphere surfaces with only BS(PEG)_9_ termination that lack the biorthogonal PEG-aldehyde functionalization as well. As such surfaces do not contain the cross-linking moieties, the negative controls exhibit poor supported bilayer coverage upon liposome fusion and/or poor, noncontinuous lipid retention (shown for example in Figure S5).

### Fluorescence Flow Cytometry Analysis of Relative Surface Density of Reactive PEG-Aldehydes and Nonspecific Binding Levels

In order to compare the relative density of surface PEG-aldehyde for supported biomembrane tethering, a portion of PEG-aldehyde-terminated beads was set aside and labeled with hydrazine containing FTSC-5 for analysis of aldehyde functionalization with flow cytometry and CLSM. The FTSC-5 mean channel fluorescence per microsphere was obtained from ∼10,000 microspheres from three independent samples. The Bangs Laboratories quantum fluorescein MESF kit was used in the quantitation of fluorescence intensity in Molecules of Equivalent Soluble Fluorochrome (MESF) units. The MESF FITC beads allowed us to establish a flow cytometry calibration curve using five standard polymeric particles with a known number of loaded fluorophores, giving rise to a range of five peaks in the FL1 channel of verified average intensity (provided by Bangs Laboratories). All cytometry data sets measured included this calibration method, allowing for a comparison of data sets conducted on different days. We analyzed the proteolipobeads along with the negative controls separately and recorded each sample’s FITC cytometry fluorescence intensity peak. Finally we used the calibration plot to determine the MESF value that corresponded to each peak. The flow cytometry was conducted using a BD Biosciences FACSCalibur flow cytometer with 488 nm excitation and mean channel fluorescence in the FL1 channel (500–550 nm) as the readout.

To quantify the nonspecific binding levels of the lipobead substrates, the labeled monoclonal anti-FLAG-FITC was used at a concentration of 1 μg/mL. The flow cytometry was conducted with 488 nm excitation, and mean channel fluorescence in the FL1 channel (500–550 nm) was the readout of the nonspecific binding. The FITC mean channel fluorescence per microsphere was obtained from ∼10,000 microspheres from three independent samples.

### Sizing of Multimarm-PEGs in Solution with Dynamic Light Scattering

Dynamic light scattering of multiarm PEG-amine tethering polymers in solution was carried out using a Malvern Zetasizer at 25 °C, obtaining size distribution intensity histograms, shown in Figure S1 of the Supporting Data. All measurements were carried out in disposable sizing cuvettes at 1 mg/mL in 0.2 μm-filtered PBS buffer. PEG samples were compared with the blank containing only 0.2 μm-filtered PBS buffer. The error bars were from the peak standard deviation computed with Malvern software.

### Lipid Film Preparation

All lipid films were prepared in anhydrous chloroform, vacuum pumped-out to remove all traces of solvent, and stored at −20 °C under argon. Tethering lipids were prepared with POPC as the base lipid and 5 mol % NH_2_-C5-PE as the NH_2_-tethering lipid at the biomembrane surface. The POPC/cholesterol/PE-hexanoylamine/DiO relative mole composition was 0.75:0.198:0.05:0.002.

### Formation of Simple Unilamellar Vesicles (SUVs) and Untethered Lipobeads

For the untethered experiments, films were hydrated to a concentration of 1 mg/mL and sonicated with an ultrasonic homogenizer (Model 150 V/T, Biologics, Inc.) at 40% power for 15 min, or until the lipid solution became transparent, indicating a near-uniform suspension of 20–50 nm SUVs. 5 μm silica microparticles (1–3 mg) suspended in 20 mM filtered PBS (at 5 mg/mL) were titrated from a 1 mL pipet into the SUV suspension, administering light vortexing every 2–3 droplets. The resulting lipobeads were then pelleted with microcentrifugation and washed with 20 mM filtered PBS three times, after which they were resuspended at a contextually appropriate concentration and imaged.

### Formation of Tethered Lipobeads

For tethered experiments, the films were hydrated to a concentration of 2 mg/mL before sonication, as described above. SUVs were then functionalized with the complementary half of the conjugation moiety, Sulfo-S-Hynic, by incubating 4 mg of SUVs with 1 mg of the Sulfo-S-Hynic, (final concentrations: ∼2 mg/mL, lipids; ∼0.45 mg/mL, Sulfo-S-Hynic,) for ∼1.5 h, mixing gently, protected from light. Excess Sulfo-S-Hynic was removed by overnight incubation with a dialysis cassette immersed in 3 L of PBS, protected from light, the resulting SUV concentration being ∼2 mg/mL. 5 μm aldehyde(SB4)-PEG-amine-functionalized silica particles with various PEG-aldehyde configurations (1–3 mg) were suspended in 20 mM PBS (at 5 mg/mL) and titrated from a 1 mL pipet into the azide(S-Hynic)-SUV suspension, administering light vortexing every 2–3 droplets. The resulting cross-linking reaction mixture was then left to mix gently in the dark in 100 mM aniline in an end-overend mixer for 30 min at room temperature, followed by 1 h at 45 °C. The resulting tethered lipobeads were washed three times in PBS and characterized.

### 3D Imaging and Confocal Fluorescence Recovery after Photobleaching (FRAP)

We used a combination of structured illumination and confocal laser scanning microscopy (SIMs and CLSM) to image the supported biomembrane structures on the bead surface, and the mobility of the lipid reporter DiO in these structures was measured using confocal-FRAP. The structured illumination microscopy was conducted with a Zeiss Elyra SIM S1 microscope.^24^ The samples were also imaged using a Leica TCS SP2 AOBS confocal microscope system equipped with argon ion and HeNe lasers. A 63*×*/1.4 NA oil-immersion objective was used for all of the CLSM and SIMs images. In the CLSM studies, DiO was excited using the 488 nm line of the argon laser at 2% intensity, and images were taken with the detection window set between 500 and 615 nm; a 1024 × 1024 pixel format was used (scan speed 100 Hz, 488 nm acousto-optic tunable filter (AOTF) 2% (power %)) over 10 μm of the *z* axis. The pinhole aperture was set at an Airy value of 1.0, which was equivalent to sampling an ∼500 nm vertical z slice of the lipobead, as estimated by the axial resolution, *r*_z,confocal_ ≈ 1.4λ_em_*n*/NA^2^ (NA, numerical aperture; *n*, refractive index of water at 25 °C (1.33); λ_em_, emission wavelength (525 nm)). Subresolution polystyrene nanospheres of 40 nm diameter (FluoSpheres fluorescent microspheres, Thermo Fisher, Inc.) adsorbed onto test silica microspheres were used to monitor the resolution by examination of the point spread function and to maintain the calibration of the 3D imaging. For the CLSM data, ImageJ64 was used to make summed Z-projections of the Z stacks from a hemisphere of the PLB. Lipid coverage was obtained by examining each CLSM hemisphere of a PLB projected to a plane, and dark areas of missing DiO staining were measured and corrected for distortion effects on the area measurements considering the data as an azimuthal equidistant projection of a hemisphere, as described previously.^7^ Sets of *N* = 20 randomly selected microspheres were included in the coverage analysis. The lipid coverage percentage error bars are computed from the standard error of the mean values. The structured illumination microscopy was performed using a Zeiss ELYRA S.1 system in the DiO green channel (laser excitation 488 nm, HR Diode 488–100 mW, 2%; gain = 20, exposure = 100 ms, BP filter detection, 495–550 nm). For the PLBs, 10 μm *z*-axis image stacks were acquired in optical steps of 110 nm. Images from five rotations and five phase shifts per rotation were processed with Zeiss Zen Black software, using raw values of 16-bit image intensities to give the 3D reconstructions. For the SIMs data, ImageJ64 was used to make summed Z-projections of the Z stacks from a hemisphere of the PLBs.

Fluorescence recovery after photobleaching (FRAP) studies were carried out using the built-in protocol of the CLSM system, as we have implemented in multiple previous studies.^15−17^ The image plane was set at the equator of the bead, and a 512 pixel × 32 pixel format was used (zoom value 16, scan speed 400 Hz, 488 nm AOTF 2% (power %)). This enabled the fast imaging (0.2 s/scan point) of two equatorially opposite ends of the bead. After five prescans, a region of ∼1 μm × 1 μm on the equator of the PLB was subjected to the bleaching laser intensity (at 100% intensity) and then the recovery of fluorescence was monitored for >30 s at normal laser intensity. Data were collected for the normalized fluorescence intensity of the bleached region throughout and analyzed using Mathematica in concert with Microsoft Excel to estimate the value of the mobile fraction, α, and the effective diffusion coefficient, *D*_eff_, in μm^2^/s.^25^ The error bars are computed from the standard error of the mean values. The FRAP data were collected in conjunction with 3D imaging to ensure that the FRAP was not recorded in regions adjacent to defects or inhomogeneities in the supported biomembrane that would lead to FRAP artifacts. Sets of *N* = 15 randomly selected microspheres were included in the FRAP analysis. Data sets with artifactual recoveries due to bead shifting or motion were excluded from the analysis. Due to the nature of the CLSM confocal illumination field propagating through subequatorial regions of the supported bilayer on the microsphere above and below the observation volume, we consider these diffusivities to be only for effective comparison across this study and not as absolute diffusivity values. The microsphere architecture allows for the assessment of many assemblies as a means to do quality control of the supported bilayer biophysical properties.

In all data, error bars were computed in terms of standard error of the mean (σ/√*N*), with the exception of the DLS measurements. A one-way analysis of variance (ANOVA) was used to compare values. Significance testing was done using the ANOVA power panel in Igor Pro software (WaveMetrics, Portland, OR). Statistical significance was set at *p* < 0.05.

## Supporting Information Available

Additional details pertaining to the materials and methods (PDF)

## Abbreviations

CLSM: confocal laser scanning microscopy
PLB: proteolipobead
SIM: structured illumination microscopy
FITC: fluorescein isothiocyanate(-labeled)
FRAP: fluorescence recovery after photobleaching
FTSC-5: fluorescein-5-thiosemicarbazide
SLB: supported lipid bilayers
PEG: poly(ethylene glycol)
SUV: small unilamellar vesicle
MESF: mean equivalents of soluble fluorescence
