# Supplementary material for: Supported Biomembrane Systems Incorporating Multiarm Polymers and Bioorthogonal Tethering
Source: Langmuir. 2024 May 20;40(22):11401–10. doi: 10.1021/acs.langmuir.4c00176 (PMC11155251; doi:10.1021/acs.langmuir.4c00176)
Supplement: Supplementary file 1 — la4c00176_si_001.pdf [file la4c00176_si_001.pdf]

SUPPLEMENTARY INFORMATION

Supported Biomembrane Systems Incorporating Multi-Arm Polymers and Bioorthogonal Tethering

Jesse A. Martin[❖] Yue-Ming Li[‡§], and M. Lane Gilchrist[†❖*]
†Department of Chemical Engineering and the ❖Department of Biomedical Engineering, The City College of the City University of New York, 140th Street and Convent Avenue, New York, NY 10031. ‡Molecular Pharmacology and Chemistry Program, Memorial Sloan-Kettering Cancer Center, 1275 York Avenue, New York, NY 10065, USA. §Program of Pharmacology, Weill Graduate School of Medical Sciences of Cornell University, New York, NY 10021, USA.

Contents: Supplementary Figures.

Figure S1: Dynamic light scattering of multiarm PEG tethering polymers in solution
Figure S2: Roughness measurements of substrate FTSC-5 and PLB tether-supported bilayers
Figure S3: Tethered polymer geometric arrangements used to estimate minimum tether density and amine-terminal group separation.
Figure S4. Representative recovery curve from a FRAP experiment for 4arm20k.
Figure S5. Representative CLSM images of negative controls of tethered lipobead formation

Methods.

The supplemental section contains an extended description of further analysis and estimates of tethering densities.

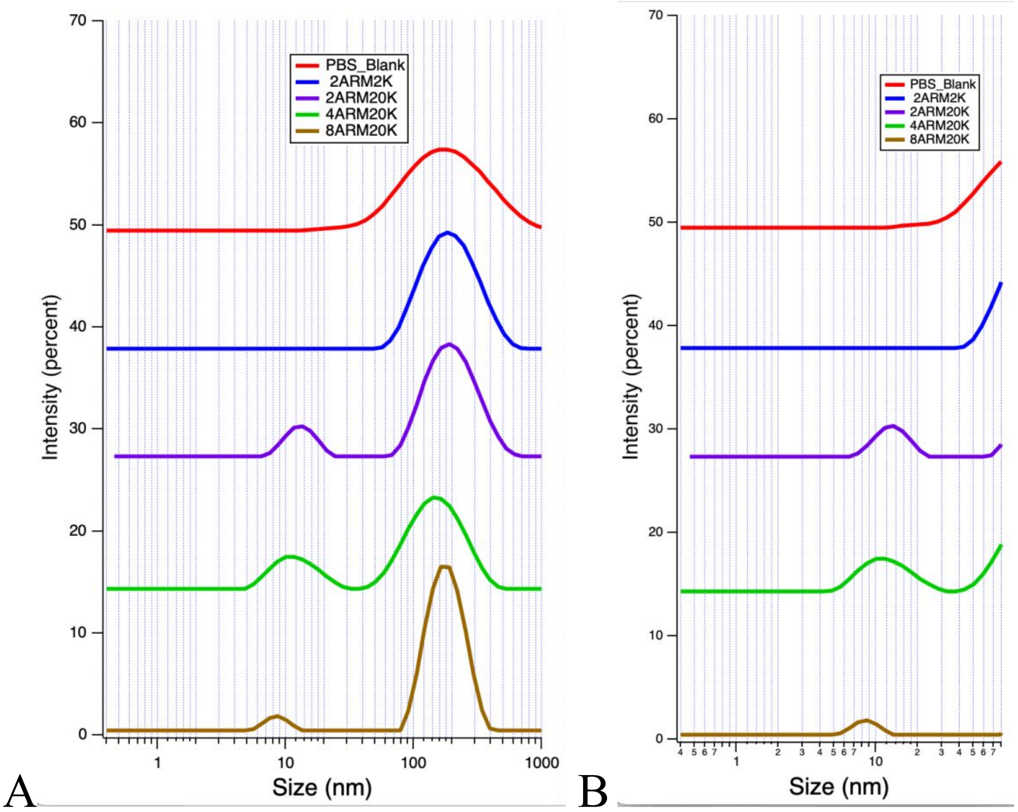

Figure S1: Dynamic Light Scattering of multiarm PEG amine tethering polymers in solution. Panel A is the size distribution intensity histograms up to 1000 nm. Panel B is the same graph magnified from 0-80 nm. The blank PBS buffer distributions are the top red trace in panels A and B. The size distributions below 15 nm are: 1) linear2k (not detected), 2) linear20k ($d_{avg,2arm20k}$ = 13.6 ± 2.8 nm). Panels B are the size distributions for the linkers 4arm20k ($d_{avg,4arm20k}$ = 12.6 ± 4.8 nm) and 4arm20k ($d_{avg,8arm20k}$ = 8.5 ± 1.5 nm), respectively. All measurements were carried out at 1 mg/ml in 0.2 micron filtered PBS buffer. The distribution width parameters are the standard deviations computed by the Malvern software. The polydispersity indexes of the PEG polymers were below 1.08 (PDI < 1.08) as given by the manufacturer Nanocs, Inc.

Figure S1 displays the Malvern Zetasizer dynamic light scattering (DLS) of multiarm PEG amine tethering polymers in solution. All samples were filtered through a 0.2 micron syringe filter prior to DLS measurements. In the left panel A is the size distribution intensity histograms up to 1000 nm is displayed. The right panel B is the same graph magnified from 0-80 nm to enhance the size distributions below 15 nm. The blank PBS buffer distributions are the top red trace in panels A and B. The size distributions below 15 nm are: 1) linear2k (not detected), 2) linear20k ($d_{avg,2arm20k}$ = 13.6 ± 2.8 nm). Panels B are the size distributions for the linkers 4arm20k ($d_{avg,4arm20k}$ = 12.6 ± 4.8 nm) and 4arm20k ($d_{avg,8arm20k}$ = 8.5 ± 1.5 nm), respectively. All measurements were carried out at 1 mg/ml in 0.2 micron filtered PBS buffer. The distribution width parameters are the standard deviations computed by the Malvern software. The average sizes given are the intensity weighted averages and are obtained directly from the size histogram peak. The standard deviation, indicative of the distribution in the peak, is also obtained directly from the histogram. For the 2arm2k (NH_2-PEG_{2000}-NH_2) case, at these conditions the Malvern Zetasizer DLS was unable to characterize the

size, even when we brought the concentration up to 5 mg/ml. The size distribution was flat and identical to the PBS buffer blank (and 1 mg/ml case) below 15 nm (5 mg/ml data not included since the size distribution was undetectable).

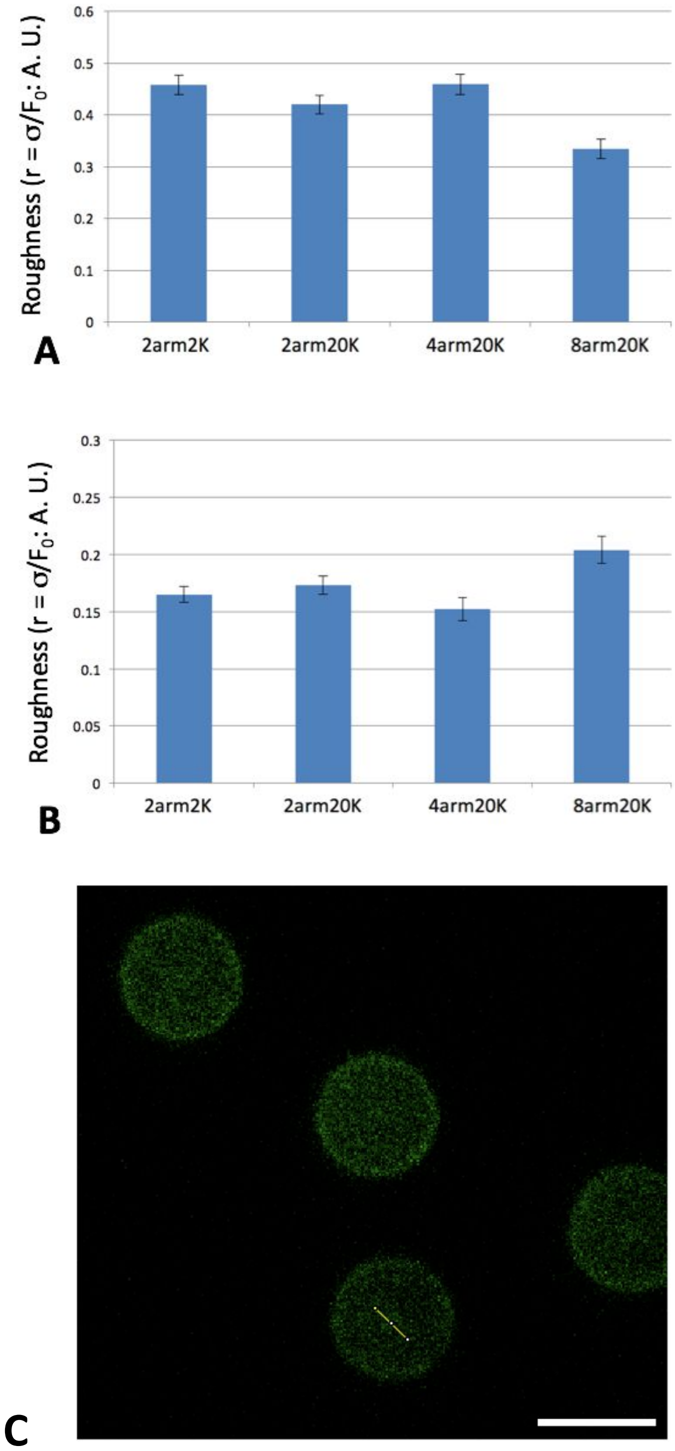

Figure S2: Roughness measurements of substrate FTSC-5 and PLB tether-supported bilayers. Panel A was obtained from the substrates labeled with FTSC-5 and panel B was obtained from the final PLBs. In panel A only the 8arm20k roughness average was significantly different from the other three ($p < 0.05$). In panel B none of the four were significantly different. Panel C displays a typical image line sampling of a 3D projection.

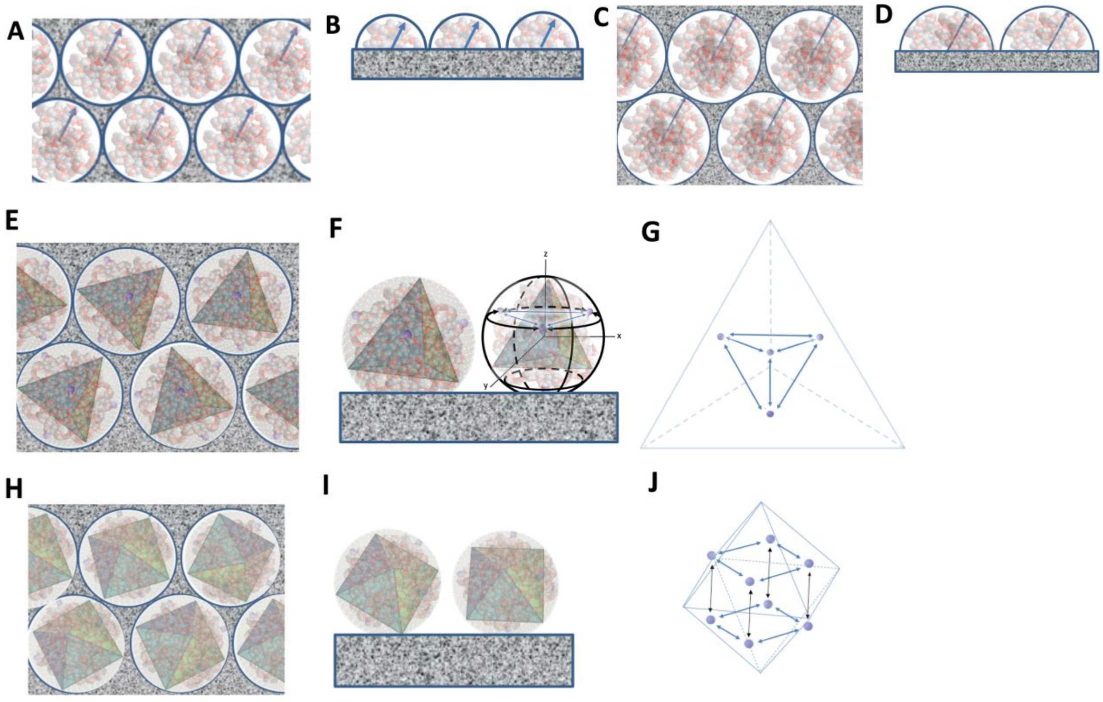

Figure S3. Idealized tethered polymer geometric arrangements used to estimate minimum tether density and amine-PEG to amine-PEG separation. In panels A (top) and B (side), the configuration of tethered linear2k would give a minimum amine-amine separation of $2xR_F$ = 7 nm in the "mushroom" regime (no overlap). Shown in panels C (top) and D (side), the configuration of tethered linear20k would give a minimum amine-amine separation of $2xR_F$ = 26.6 nm in the "mushroom" regime (no overlap). Panels E-G are schematics showing terminal amino groups positioned as PEG arms radiating out for the center relative to the faces of a tetrahedron for 4arm20k, with E from the top, F from the side and G displaying the underlying symmetry (the blue dots are the amine groups). Likewise for 8arm20k, panels H-J show schematically the idealized positioning of terminal amino groups with H from the top, I from the side and J displaying the underlying symmetry and amine-PEG separations (the blue dots are the amine groups). in panels G and J in the double sided arrows, this spatial arrangement gives the estimated closest approach of amine groups of 10.3 nm for 4arm20k-PEG and 4.9 nm for 8arm20k-PEG, considering approximate arms lengths of 6.3 and 4.4 nm for each, respectively (based on R_{hyd} from DLS).

We used idealized tethered polymer geometric arrangements to estimate the minimum tether density and amine-PEG NH_2-NH_2 separations assuming no overlap of the tethered polymers on the surface in the "mushroom" regime. In panels A (top) and B (side), the configuration of tethered linear2k would give a minimum amine-amine separation of $2xR_F$ = 7 nm in the "mushroom" regime. Likewise, as shown in panels C (top) and D (side), the configuration of tethered linear20k would give a minimum amine-amine separation of $2xR_F$ = 26.6 nm in the "mushroom" regime. Considering a circle packing fraction (0.909) and no overlap, the maximum grafting density for 2arm2k and 2arm20k would be 0.26 and 0.07 NH_2-PEG/nm^2, respectively. Panels E-G are schematics showing terminal amino groups positioned as PEG arms radiating out for the center relative to the faces of a tetrahedron for 4arm20k, with E from the top, F from the side and G displaying the underlying symmetry. Likewise for 8arm20k, panels H-J show

schematically the positioning of terminal amino groups with H from the top, I from the side and J displaying the underlying symmetry and amine terminal group separations (blue dots). Considering a circular packing fraction (0.909), the maximum grafting density at no overlap for 2arm2k and 2arm20k would be 0.02 and 0.08 NH_2-PEG/nm^2, respectively. As shown in panels G and J in the double sided arrows, this spatial arrangement gives the estimated closest approach of amine groups of 10.3 nm for 4arm20k-PEG and 4.9 nm for 8arm20k-PEG with no overlap, considering arms lengths of 6.3 and 4.4 nm for each, respectively (based on R_{hyd}) from the DLS measurement. These estimates are based on assuming that the amino groups positioned on the end of PEG arms radiating out for the center relative to projecting out to the center of the faces of a tetrahedron (4arm20k) and octahedron (8arm20k), outlined schematically in figure S3 panels G and J (amines are blue dots).

Fluorescence Recovery After Photobleaching (FRAP). Calculation of effective diffusion coefficients, D_{eff}, of PLB membrane-integrated lipid tracers (DiO) was performed by fitting raw FRAP data to the following formalism described by Klonis et al [1]:

Where:

F(t) = Fluorescence intensity of the bleached spot at time t after bleaching

$$\frac{F(t)}{F_p} = \alpha \sum_{n=0}^{\infty} \left[\left(\frac{(-K)^n}{n!} \right) \left(\frac{1}{1+n(1+2t/\tau_D)} \right) \right] + (1-\alpha)\frac{F_o}{F_p}$$

F_p = Prebleach intensity

F_o = Intensity immediately after bleaching

Mobile fraction, $\alpha = \frac{F_\infty - F_o}{F_p - F_o}$

K is a parameter related to the degree of bleaching by

$$\frac{F_o}{F_p} = \frac{1-e^{-K}}{K}$$

and τ_D is the characteristic diffusion time, related to diffusion coefficient by

$D_{eff} = \frac{\omega^2}{4\tau_D}$ where ω is the Gaussian radius of the bleaching laser, taken to be 1 µm.

Prior to data fitting, raw time-lapse fluorescence curves of bleach and reference regions drawn by hand in ImageJ (necessarily using a reference region from membrane segments opposite to the bleach region) were tabulated in IGOR Pro, where normalized recovery curves were generated. First, reference region curves were fitted as exponential decays. Normalizing fluorescence recovery was done in two steps: (1) Dividing bleach region signal intensities by fitted-reference region signal intensities, and (2) Setting pre-bleach signal intensities to 1 by dividing the result of

step (1) by the pre-bleach intensity generated in step (1). Finally, normalized fluorescence was truncated to include only post-bleach frames up to 6 seconds. This data was exported to Microsoft Excel, where fitting was performed.

F_o/F_p was first calculated using the averages of two pre-bleach and three post-bleach intensities. K was solved with the relation written above using the Solver function in Excel. Fitting was

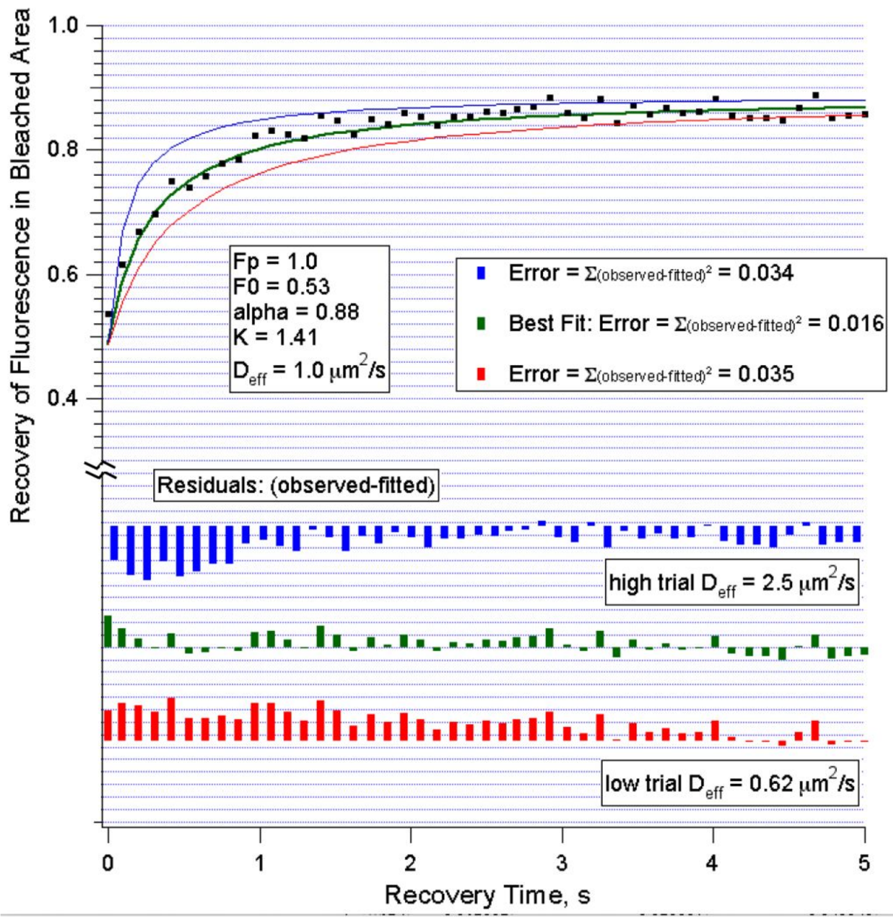

Figure S4. Representative recovery curve from a FRAP experiment for 4arm20k. Bleach region profiles were normalized using reference regions from the same PLB membrane, located opposite to the bleach region in equatorial confocal z-slices.

performed by running Solver with α and τ_D named as changeable cells and minimizing the root-mean-square deviation (RMSD) between experimental and fitted intensity values. RMSD is defined as

$$RMSD = \sqrt{\frac{\sum_i (I_{exp,i} - I_{fit,i})^2}{N}}$$

where $I_{exp,i}$ is the experimental normalized intensity at time point i, $I_{fit,i}$ is the fit normalized intensity at time point i, and N is the total number of time points.

Figure S4 displays representative FRAP data from a tethered PLB sample with the fit and calculated K, mobile fraction, τ_D and D_{eff} included.

References

[1] Klonis, N.; Rug, M.; Harper, I.; Wickham, M.; Cowman, A.; Tilley, L. *Eur Biophys J* **2002**, *31*, 36.

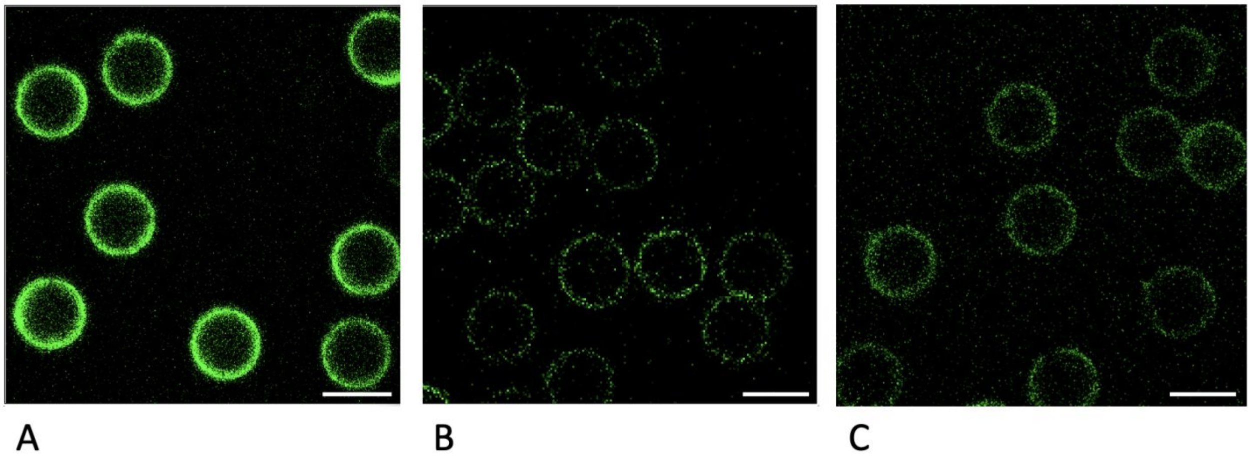

Figure S5. Representative CLSM images of negative controls of tethered lipobead formation. The left panel is an equatorial CLSM z section of tethered lipobeads that contain the 4arm20k bioorthogonal coupling as desired and good DiO mobility via FRAP (Figure S4). Panel B is the equatorial z section of $BS(PEG)_9$ terminated microspheres and panel C is the equatorial z section of $BS(PEG)_9$-PEG2k-NH2 terminated microspheres. In panels B and C, the Sulfo-S-HyNic-functionalized liposome fusion was unsuccessful as the microsphere surface lacks aldehyde functionalization for bioorthogonal conjugation. These negative controls exhibit poor supported bilayer coverage upon liposome fusion, and/or poor, non-continuous lipid retention leading to inconsistent and inadequate supported biomembranes and insufficient signal intensity for quality 3D imaging.
